# Development of the Tendency to Use Emotion Regulation Strategies and Their Relation to Depressive Symptoms in Chinese Adolescents

**DOI:** 10.3389/fpsyg.2016.01222

**Published:** 2016-08-22

**Authors:** Liyang Sai, Sichen Luo, Anne Ward, Biao Sang

**Affiliations:** ^1^Institutes of Psychological sciences, Hangzhou Normal University, HangzhouChina; ^2^Key Laboratory of Brain Functional Genomics (MOE & STCSM), School of Psychology and Cognitive Science, East China Normal University, ShanghaiChina; ^3^Center for Cognition and Brain Disorders, Hangzhou Normal University, HangzhouChina; ^4^Zhejiang Key Laboratory for Research in Assessment of Cognitive Impairments, HangzhouChina; ^5^Department of Psychology, Zhejiang Normal University, JinhuaChina; ^6^Department of Psychology, Northwestern University, Evanston, ILUSA

**Keywords:** emotion regulation, cognitive reappraisal, expressive suppression, adolescence

## Abstract

The process model of emotion regulation posits that the tendency to use cognitive reappraisal is associated with positive outcomes (e.g., greater positive emotion) while the tendency to use expressive suppression is associated with adverse outcomes (e.g., greater negative emotion). Many studies using adult samples support this theory. However, the development of the tendency to use cognitive reappraisal/expressive suppression and how these tendencies relate to depressive symptoms in adolescents remain unclear. To address these questions, 639 Chinese adolescents aged 12–18 years old were asked to report their tendency to use cognitive reappraisal/expressive suppression as well as their depressive symptoms. General linear model multivariate analysis of variance showed a statistically significant age effect for the tendency to use emotion regulation strategies. Further analysis found that these adolescents reported using less expressive suppression as age increased, while there was no age effect for the tendency to use cognitive reappraisal. Moreover, linear regression analysis revealed that the tendency to use cognitive reappraisal in daily life negatively influenced depressive symptoms, while the tendency to use expressive suppression in daily life positively influenced depressive symptoms. These findings provide evidence that support the development of emotion regulation strategies in childhood and adolescence.

## Introduction

Adolescence is a period of emotional “storm and stress” (Casey et al., 2010). Teens experience more extreme emotions and more variable mood states than adults in daily life (Larson et al., 2002), and are also vulnerable to emotion-related disorders such as anxiety and depression (Allen and Sheeber, 2008). Although these emotional characteristics can be partly attributed to changes in biological, cognitive, social, and emotional domains, recent evidence suggests that emotion regulation deficits also play an important role (Silk et al., 2003; Betts et al., 2009). Thus, it is important for us to understand the development of emotion regulation in adolescence.

Emotion regulation refers to the set of processes by which we modify the experience and expression of our emotions (Gross, 1998b). Though there are many emotion regulation strategies, the process model of emotion regulation distinguishes emotion regulation strategies as *antecedent-focused* and *response-focused* (Gross, 1998a). Antecedent-focused strategies refer to strategies adopted before the emotion-response tendencies have become fully activated while response-focused strategies refer to those adopted once an emotion has been fully generated. Two commonly used strategies—cognitive reappraisal and expressive suppression—have been extensively operationalized within this model. Cognitive reappraisal alters the trajectory of emotional responses by changing the meaning of the situation and is an example of an antecedent-focused strategy. Expressive suppression involves inhibiting behaviors (e.g., facial expressions) associated with emotional responding is an example of a response-focused strategy (Gross and John, 2003; John and Gross, 2004).

Based on the process model of emotion regulation, most studies have examined individual differences in the tendency to use cognitive reappraisal/expressive suppression and their relationship to psychological functioning (Gross and John, 2003; Butler et al., 2007; Soto et al., 2011). For example, it has been demonstrated in adults that the tendency to engage in cognitive reappraisal is associated with increased positive emotion, decreased negative emotion, better interpersonal functioning, and increased well-being and life satisfaction. Conversely, it has also been shown in adults that the tendency to use expressive suppression is associated with increased negative emotion, decreased positive emotion, worse interpersonal functioning, and decreased well-being and life satisfaction. These findings suggest that cognitive reappraisal is a healthier emotion regulation strategy than expressive suppression (John and Gross, 2004).

Based on these findings, a few studies have investigated the development of the tendency to use cognitive reappraisal/expressive suppression in adolescence (e.g., Gullone et al., 2010; Lantrip et al., 2016; Gómez-Ortiz et al., 2016). Gullone et al. (2010) and Gómez-Ortiz et al. (2016) found that adolescents reported using less cognitive reappraisal and expressive suppression with increasing age. This decreased use of expressive suppression seems to indicate that adolescents learn that expressive suppression is not an adaptive emotion regulation strategy with age; however, it is hard to interpret the decreased use of cognitive reappraisal since the adolescents were expected to learn to use healthier emotion regulation approaches (John and Gross, 2004). In another study, Lantrip et al. (2016) found no age effects for cognitive reappraisal or expressive suppression. However, it should be noted that their sample size was small (*N* = 70). Given these mixed findings, the development of the tendency use to cognitive reappraisal/expressive suppression in adolescence is still unclear.

Depression is characterized by prevalent negative emotion, and has a great adverse impact on individuals’ emotional, social, and occupational functioning (American Psychiatric Association, 2013). Adolescence is a critical period for the onset of depressive symptoms, (Allen and Sheeber, 2008) and the prevalence of depressive disorder is around 5.6% in adolescents compared to only 2.8% in children (Jane Costello et al., 2006). Longitudinal studies also show that an episode of depression in adolescence is a risk factor for subsequent episodes (Birmaher et al., 1996). Thus, it is important to identify risk factors that could lead to the development of this disorder, as well as protective factors that could contribute to the prevention and treatment of adolescent depression. Since depression is characterized by prevalent unpleasant emotions, it is not surprising that researchers have attributed the maintenance of such emotional experiences to emotion regulation deficits (for a review, see Sheppes et al., 2015). Although many researchers have linked emotion regulation with depressive symptoms, only two studies have investigated the relationships between cognitive reappraisal/expressive suppression tendencies and depressive symptoms in adolescence. For example, Betts et al. (2009) found that, compared to a control group, adolescents (12–16 years) with high scores on a self-report depressive symptomatology questionnaire were more likely to endorse using expressive suppression and less likely to report using cognitive reappraisal. In addition, Larsen et al. (2013) found that the tendency to use expressive suppression was significantly correlated with depressive symptoms in early adolescence (~13 years). These findings suggest that the tendency to engage in cognitive reappraisal protects against depressive symptoms while the tendency to use expressive suppression increases risk for depressive symptoms in adolescence.

### Culture and Suppression

Though the above mentioned studies offer insight into the development of cognitive reappraisal and how this construct relates to depressive symptoms, it is important to note that they were based on Western samples. Recent evidence suggests that culture has a great impact on the tendency to use expressive suppression and influences how it relates to psychological functioning. For example, Butler et al. (2007) found that the tendency to use expressive suppression was associated with greater negative emotion for women endorsing strong European values while it was associated with less negative emotion for women with bicultural values (e.g., European and Asian values). Furthermore, Soto et al. (2011) found that the tendency to engage in expressive suppression was associated with less life satisfaction and increased depressed mood in European Americans, while there was no correlation between expressive suppression and life satisfaction or depressed mood among Chinese participants. These findings suggest that expressive suppression is not as maladaptive in Asian culture as in Western European cultures. However, these findings were based on adult samples, and it remains unclear how the development of cognitive reappraisal/expressive suppression strategies relate to depressive symptoms in East Asian adolescents.

To address these issues, we investigated the development of the tendency to use cognitive reappraisal/expressive suppression in Chinese adolescents (12–18 years) using Emotion Regulation Questionnaire (ERQ). We also explored the associations between the tendency to use cognitive reappraisal/expressive suppression strategies and depressive symptoms. Since prior work has produced mixed findings (Gullone et al., 2010; Gómez-Ortiz et al., 2016; Lantrip et al., 2016), we did not make predictions about the development of cognitive reappraisal/expressive suppression. Regarding their impact on depressive symptoms, we expected frequent cognitive reappraisal use to be associated with reduced depressive symptoms (Betts et al., 2009). However, since no correlation between expressive suppression use and depressive symptoms were found in Chinese adults (Soto et al., 2011), we did not expect to find a significant relationship between expressive suppression tendency and depressive symptoms with our adolescent sample.

## Materials and Methods

### Ethical Approval

All procedures performed in studies involving human participants were in accordance with the ethical standards of the institutional and/or national research committee and with the 1964 Helsinki declaration and its later amendments or comparable ethical standards.

### Participants

Participants in the study (*N* = 639, 345 female) were adolescents ranging from 12 to 18 years of age who were randomly selected from three schools (21 classrooms) in the Jinhua city, China. All children were of Han nationality, which is the predominant ethnic group in China. The sample was divided into seven age groups: 12 years (*N* = 62; mean age = 12.31 years; *SD* = 0.51), 13 years (*N* = 78; mean age = 13.30 years; *SD* = 0.31), 14 years (*N* = 103; mean age = 14.29 years; *SD* = 0.34), 15 years (*N* = 128; mean age = 15.41 years; *SD* = 0.32), 16 years (*N* = 101; mean age = 16.44 years; *SD* = 0.29), 17 years (*N* = 82; mean age = 17.31 years; *SD* = 0.34), 18 years (*N* = 85; mean age = 18.30 years; *SD* = 0.32). Informed consent was obtained from all participants and the study was approved by the Ethics Committee of Zhejiang Normal University.

### Measures

The ERQ was administered to gauge adolescents’ proclivity to engage in cognitive reappraisal and expressive suppression in daily life (Gross and John, 2003). This scale consists of 10 items, six of which assess cognitive reappraisal (e.g., “I control my emotions by changing the way I think about the situation I’m in”), and four of which assess expressive suppression (e.g., “I control my emotions by not expressing them”). Ratings were made on a 1 (strongly disagree) to 7 (strongly agree) scale. A Chinese version of this questionnaire that has been validated for both adults and adolescents was used (e.g., Wang et al., 2007). In the present study, the Cronbach’s alpha coefficient was 0.72 for the reappraisal subscale and 0.70 for the suppression subscale.

Adolescents’ depressive symptoms were measured using a Chinese version of the Center for Epidemiological Studies Depression (CES-D) inventory, which consists of 20 items (Radloff, 1977). For example, “I was bothered by things that usually do not bother me.” Participants were asked to indicate how frequently in the past week each depressive symptom occurred using four-point scale [1: occasionally (less than one day); 4: most of the time (five to seven days)]. Cronbach’s alpha for the CES-D was 0.84 in the present study.

### Statistical Analysis

First, to examine age-related difference in cognitive reappraisal/expressive suppression tendencies, a two-way multivariate analysis of variance (MANOVA) was performed with age group and gender as between-subjects variables (e.g., Pepe and Addimando, 2014).

Second, to examine the relationships between cognitive reappraisal/expressive suppression tendencies and depressive symptoms, Pearson correlations were conducted with age, gender, cognitive reappraisal/expressive suppression, and depressive symptoms. Furthermore, a hierarchical linear regression was conducted with depressive symptoms scores as the dependent variable. Age (continuous variable) was entered into the model on the first step, followed by gender on the second step, and finally cognitive reappraisal scores and expressive suppression scores on the third step.

## Results

### Development of Tendency to Use Cognitive Reappraisal/Expressive Suppression

**FIGURE 1:**
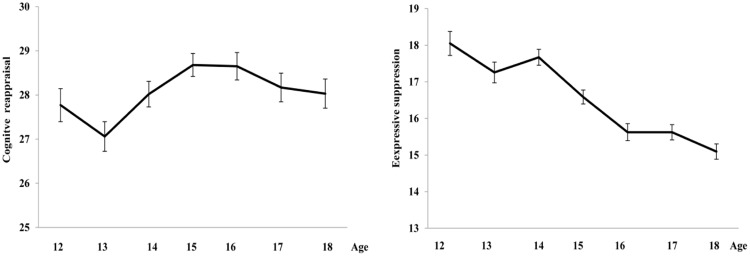
**Development of cognitive reappraisal and expressive suppression in Chinese adolescence**.

The result of MANCOVA revealed a statistically significant main effect of age group (Wilks’ λ = 0.94, *F* = 3.12, *p* < 0.001, $\eta_{p}^{2}$ = 0.030), a marginally statistically significant main effect of gender (Wilks’ λ = 0.99, *F* = 2.65, *p* = 0.07, $\eta_{p}^{2}$ = 0.008), and no statistically significant interaction between age and gender (Wilks’ λ = 0.97, *F* = 1.42, *p* = 0.15, $\eta_{p}^{2}$ = 0.01). Univariate analyses revealed that the effect of age group on expressive suppression scores was statistically significant [*F*(6,625) = 4.64, *p* < 0.001, $\eta_{p}^{2}$ = 0.04], while the effect on cognitive reappraisal scores was not [*F*(6,625) = 0.7, *p* = 0.60]. *Post hoc* tests for expressive suppression scores indicated that the tendency to use expressive suppression decreased at around 15 years old and then stabilized from 16 until 18 years old (see **Figure 1** and **Table 1**).

**Table 1:** *Post hoc* comparison for expressive suppression scores between age groups.

*p*	12 years	13 years	14 years	15 years	16 years	17 years
12 years						
13 years	0.26					
14 years	0.58	0.50				
15 years	0.037*	0.36	0.077			
16 years	0.003**	0.05*	0.005**	0.21		
17 years	0.001**	0.024*	0.002**	0.11	0.73	
18 years	0.000**	0.006**	0.000**	0.033*	0.39	0.62

*p < 0.05, **p < 0.01.

### Tendency to Use Cognitive Reappraisal/Expressive Suppression Influences Depressive Symptoms in Chinese Adolescents

**Table 2** displays the mean, standard deviation, range, and skew of each variable. **Table 3** shows Pearson correlation coefficients among the variables. Depression symptoms had a statistically significant negative correlation with cognitive reappraisal scores (*r* = -0.18, *p* < 0.001), and had no statistically significant correlation with expressive suppression scores (*r* = 0.01, *p* > 0.05).

**Table 2:** Descriptive statistic of each variable.

	*M*	*SD*	Range	Skewness
Age	15.46	1.88	12–18	-0.10
Cognitive reappraisal	28.17	5.92	8–42	-0.07
Expressive suppression	16.51	4.54	6–28	0.15
Depression	35.85	8.83	20–68	0.87

**Table 3:** Zero-order correlations between variables.

	Age	Gender	Cognitive reappraisal	Expressive suppression
Age				
Gender				
Cognitive reappraisal	0.06	–0.04		
Expressive suppression	–0.21**	–0.9*	0.25**	
Depression	0.08	–0.10*	–0.18**	0.01

*p < 0.05, **p < 0.01.

**FIGURE 2:**
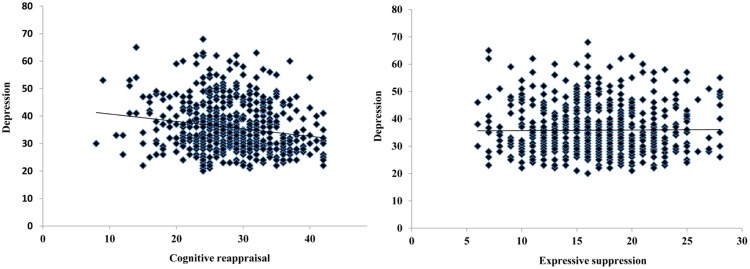
**Correlations between cognitive reappraisal/expressive suppression and depression symptoms**.

To control for possible effects of age and gender on the relationships between cognitive reappraisal/expressive suppression and depressive symptoms, a hierarchical linear regression was conducted with depression symptoms scores as the predicted variable and age in years as a continuous predictor variable. Age was entered into the model on the first step, followed by gender on the second and finally cognitive reappraisal scores and expressive suppression scores on the third. The first model was marginally significant [*F*(1,632) = 12.15, Δ*R*^2^ = 0.006, *p* = 0.053] and the second was statistically significant [Δ*F*(1,631) = 5.51, Δ*R*^2^ = 0.009, *p* = 0.019]. Finally, after partialling out the effects of age and gender, the third step was also significant [Δ*F*(2,629) = 14.81, Δ*R*^2^ = 0.04, *p* < 0.0001], suggesting that emotion regulation strategy use in everyday life is significantly related to Chinese adolescents’ depressive symptoms scores. Furthermore, both cognitive reappraisal and expressive suppression significantly contributed in the model (β = -0.32, *t* = -5.38, *p* < 0.0001, part correlation = -0.21; β = 0.18, *t* = 2.30, *p* = 0.02, part correlation = 0.072). These results indicate that the frequent use of cognitive reappraisal and the infrequent use of expressive suppression are associated with lower depressive symptoms (see **Figure 2**).

## Discussion

The present study examined the development of the tendency to use cognitive reappraisal/expressive suppression emotion regulation strategies and how these strategies relate to depressive symptoms in a large sample of Chinese adolescents. Results showed less expressive suppression use with age and no age effect for cognitive reappraisal use. Furthermore, after controlling for age and gender, cognitive reappraisal use negatively influenced depressive symptoms while expressive suppression use positively influenced depressive symptoms. These results are discussed in detail below.

We found that the tendency to use cognitive reappraisal did not increase with age. This finding is in line with another recent study which also reported no difference in cognitive reappraisal use between older and younger adolescents (Lantrip et al., 2016). Since cognitive reappraisal is an adaptive emotion regulation strategy (Gross and John, 2003), it would make sense that adolescents should learn to use this tool more in their daily life with age. However, some recent studies have suggested that children as young as six can use cognitive reappraisal to regulate their emotions (Silvers et al., 2014; Giuliani and Pfeifer, 2015). Thus, it is possible that the use of cognitive reappraisal for emotion regulation purposes is beginning earlier in development than originally thought, but becomes well-developed in early adolescence. Therefore, we did not find the age effect for cognitive reappraisal use in adolescence.

We found that expressive suppression use decreased as age increased, stabilizing at 15 years. Although some studies using adult samples have shown that expressive suppression in East Asian cultures was not associated with negative psychological functioning (Soto et al., 2011) and was instead associated with decreased negative emotion [Butler et al. (2007), study 1], it is possible that expressive suppression use was associated with adverse outcomes in Chinese adolescents. This notion is supported by a recent finding suggesting that expressive suppression use was positively correlated with depressive symptoms in a late Chinese adolescent population (Zhao and Zhao, 2015). Thus, Chinese adolescents reported less use of expressive suppression with age. Our result is consistent with result of Gullone et al. (2010), who also found that older adolescents reported using less expressive suppression compared to younger adolescents in Western culture. However, previous studies have found cultural differences in expressive suppression tendencies between adults from East Asian and Western cultures. For example, Soto et al. (2011) found that Chinese participants reported using more expressive suppression than Western participants. Instead, our results suggest that the development of the tendency to use expressive suppression is similar across both cultures. In the future, this should be further examined by including adolescents from both East Asian and Western cultures, within a single study.

Furthermore, we found that increased cognitive reappraisal use was associated with decreased depressive symptoms. This is consistent with Zhao and Zhao (2015), who also found a negative correlation between cognitive reappraisal use and depressive symptoms in Chinese late adolescents with a limited age range (i.e., 16–18 years). Together, these findings suggest that cognitive reappraisal can protect again depressive symptoms in Chinese adolescents. We also found that increased expressive suppression use was associated with increased depressive symptoms, which contradicts the findings of Soto et al. (2011), who found that expressive suppression did not correlate with depressive symptoms in Chinese adults (Soto et al., 2011). However, our findings were consistent with those of another study, which also found that the tendency to use expressive suppression was positively related to depressive symptoms in Chinese late adolescents (Zhao and Zhao, 2015). Together, these findings suggest that expressive suppression use is positively associated with depressive symptoms in Chinese adolescents, but that this correlation is no longer evident by adulthood. This may be because Chinese adolescents are not very efficient at using expressive suppression to regulate their emotions, which may lead to more negative affect, and ultimately result in increased depressive symptoms. However, with more use, Chinese adults can successfully employ expressive suppression to regulate their emotions (e.g., Murata et al., 2013), thus, expressive suppression use is not related to depressive symptoms in Chinese adults (Soto et al., 2011). Future studies should directly test this hypothesis by examining the implementation of expressive suppression in Chinese adolescents.

It should be noted that studies using European participants have typically found that expressive suppression use is strongly related to depressive symptoms, while cognitive reappraisal use is weakly related to depressive symptoms (for meta-analysis, Aldao et al., 2010). On the contrary, the present study found that the relationship between the tendency to use expressive suppression and depressive symptoms using a Chinese sample was weak (β = 0.18, *t* = 2.30, *p* = 0.02, part correlation = 0.072), while the relationship between the tendency use to cognitive reappraisal and depressive symptoms was strong (β = -0.32, *t* = -5.38, *p* < 0.0001, part correlation = -0.21). This result could be because East Asian culture encourages the use of expressive suppression, which may make individuals from this culture more capable at using this regulation technique to modify their negative emotions than individuals from Westen culture, thus resulting in less depressive symptoms (Murata et al., 2013; Yuan et al., 2015). However, cross-cultural studies are needed to directly test this hypothesis.

A limitation of the present study was its cross-section design, which was used to examine the development of cognitive reappraisal/expressive suppression tendencies and their relationship to depressive symptoms. However, this approach prevented us from investigating within-individual developmental changes. Future work should use a longitudinal design to further examine this question.

## Author Contributions

LS: experiment design, data analysis, and write the paper. SL: collect the participants and data analysis. AW: write the paper. BS: experiment design and write the paper.

## Conflict of Interest Statement

The authors declare that the research was conducted in the absence of any commercial or financial relationships that could be construed as a potential conflict of interest.
